# The mTORC1-Signaling Pathway and Hepatic Polyribosome Profile Are Enhanced after the Recovery of a Protein Restricted Diet by a Combination of Soy or Black Bean with Corn Protein

**DOI:** 10.3390/nu8090573

**Published:** 2016-09-20

**Authors:** Claudia C. Márquez-Mota, Cinthya Rodriguez-Gaytan, Pauline Adjibade, Rachid Mazroui, Amanda Gálvez, Omar Granados, Armando R. Tovar, Nimbe Torres

**Affiliations:** 1 Departamento de Fisiología de la Nutrición, Instituto Nacional de Ciencias Médicas y Nutrición Salvador Zubirán, Ciudad de México 14080, Mexico; claudia.qa@gmail.com (C.C.M.-M.); citzelg@hotmail.com (C.R.-G.); ograpo@yahoo.com (O.G.); tovar.ar@gmail.com (A.R.T.); 2 Centre de Recherche du Chu de Québec, Département de Biologie Moléculaire, Biochimie Médicale et Pathologie, Faculté de Médicine, Université Laval, Québec City, QC G1V 0A6, Canada; pauline.adjibade@gmail.com (P.A.); Rachid.Mazroui@crchudequebec.ulaval.ca (R.M.); 3 Facultad de Química, Universidad Nacional Autónoma de México, Ciudad de México 04510, Mexico; galvez@unam.mx

**Keywords:** soy protein, black bean protein, SNAT2, mTORC1-signaling pathway, polysome profiling, protein rehabilitation

## Abstract

Between 6% and 11% of the world’s population suffers from malnutrition or undernutrition associated with poverty, aging or long-term hospitalization. The present work examined the effect of different types of proteins on the mechanistic target of rapamycin (mTORC1)-signaling pathway in: (1) healthy; and (2) protein restricted rats. (1) In total, 200 rats were divided into eight groups and fed one of the following diets: 20% casein (C), soy (S), black bean (B), B + Corn (BCr), Pea (P), spirulina (Sp), sesame (Se) or Corn (Cr). Rats fed C or BCr had the highest body weight gain; rats fed BCr had the highest pS6K1/S6K1 ratio; rats fed B, BCr or P had the highest eIF4G expression; (2) In total, 84 rats were fed 0.5% C for 21 day and protein rehabilitated with different proteins. The S, soy + Corn (SCr) and BCr groups had the highest body weight gain. Rats fed SCr and BCr had the highest eIF4G expression and liver polysome formation. These findings suggest that the quality of the dietary proteins modulate the mTORC1-signaling pathway. In conclusion, the combination of BCr or SCr are the best proteins for dietary protein rehabilitation due to the significant increase in body weight, activation of the mTORC1-signaling pathway in liver and muscle, and liver polysome formation.

## 1. Introduction

A deficiency of protein and energy causes a type of malnutrition called protein-energy malnutrition (PEM), which affects children, elderly people and hospitalized patients [1,2] and accounts for six million deaths annually [3]. Numerous studies have demonstrated the extent of malnutrition in hospitals, with a prevalence reaching 50% depending on the patient population and the criteria of malnutrition [4]. According to the Food and Agriculture Organization of the United Nations (FAO), approximately 795 million people are undernourished globally and, although the number of undernourished people has decreased in recent years, this remains an important health issue [5]. Several approaches have been used to improve the nutritional status of people with malnutrition in developing countries. Two approaches are dietary enrichment [6] and oral protein calorie supplementation [7]. In the case of PEM, the main approach used to improve body weight and recovery is the use of a combination of vegetable proteins, such as corn, beans, sorghum, rice, soy and peanuts, among others [8]. It has been widely reported that the combination of cereals and legumes complement the limiting amino acids in each source [8,9,10]. Although several studies have demonstrated the effect of dietary protein, mainly casein or whey [11,12], on growth and protein synthesis [13,14,15], less is known about the effect of different dietary protein sources or combinations of vegetable proteins to activate liver and muscle mTORC1 (mechanistic target of rapamycin)-signaling pathway. Clinical and consumer market interest is increasingly directed toward the use of plant-based proteins as dietary components to increase protein synthesis; however, there is controversy about whether the ingestion of a plant-based diet results in lower protein synthesis compared to animal-derived proteins. This could be attributed to the lower digestibility of some plant-based sources due to the low content of some essential amino acids such as methionine in legumes and lysine in cereals. Furthermore, most plant proteins have a relatively low leucine content (with the exception of corn), which may further reduce protein synthesis when compared with animal proteins [16]. However, diets as consumed inevitably consist of mixtures of proteins, which improve the quality of the protein by complementing the limiting amino acids in each source. Most studies have assessed muscle protein synthesis after the ingestion of free amino acids, milk, whey or casein [12]. In contrast, few studies have assessed the impact of plant-based protein ingestion on mTORC1-signaling pathway involved in protein synthesis. It is surprising that only soy protein [17] and wheat protein [11] have been studied for its postprandial muscle protein synthesis. Combining proteins is of high priority in developing countries to improve the quality of the vegetable proteins and the lower cost than animal proteins [18] and for decreasing the risk of cardiovascular disease and type 2 diabetes [19]. From the standpoint of global sustainability, plant-based foods are proposed to be advantageous over animal-based foods [16]. It has been suggested that the production of plant-based foods requires less water, land, and energy.

It has long been known that starvation or lack of nutrients influences protein synthesis rates in mammalian tissues and cells [20,21]. Nutrient availability, growth factors and the energy of the cell are important for the regulation of the translational machinery linked to the mTORC1 (mechanistic target of rapamycin) pathway [20,22]. mTORC1 is a serine/threonine protein kinase that belongs to the family of phosphatidylinositide-3 kinase related kinases (PI3KK). Pharmacological and genetic studies have demonstrated that mTORC1 activation increases cell growth in diverse organisms from yeast to humans [22,23]. Amino acid transporters have been considered as the amino acid sensor and play a role in the activation of the mTORC1-signaling pathway, particularly SNAT2 (small neutral amino acid transporter 2), a member of the SLC38 family that have a dual activity of transporter and transceptor [24]. SNAT2 transports alanine, asparagine, cysteine, glutamine, histidine, methionine, proline and serine [25]. The activity of SNAT2 allows the accumulation of intracellular amino acids which facilitates exchange uptake of amino acids through transporters such as the l-type amino acid transporter 1 (LAT1) to increase the transport of branched chain amino acids into the cell leading to the activation of mTORC1 [24,26]. Signaling through mTORC1 is activated by amino acids [27], with the branched-chain amino acid leucine playing a major role [28]. The activated mTORC1 phosphorylates substrates such as eukaryotic initiation factor 4E-binding protein 1 (4E-BP1) and p70 ribosomal S6 kinase (S6K) [29]. The phosphorylation of 4E-BP1 prevents its binding to the cap-binding protein eIF4E, enabling it to participate in the formation of the eIF4F complex, which is required for the initiation of cap-dependent translation. Through a variety of effectors, activation of S6K1 leads to an increase in mRNA biogenesis, as well as translational initiation and elongation [30]. On the contrary, when there is a scarcity of amino acids, GCN2 (general control nonderepressible 2) is activated and phosphorylates eIF2α (eukaryotic initiation factor 2 alpha) leading to the general inhibition of protein synthesis [31]. Few studies have assessed postprandial liver and muscle protein synthesis after the ingestion of plant proteins or combinations of plant proteins in healthy and after a protein restricted period. Therefore, the purpose of this study was: (1) to determine the effect of different dietary vegetable proteins such as pea, sesame, spirulina, corn, black bean, soy protein or the combination of proteins on weight gain, biochemical parameters and the ability to activate the mTORC1 pathway in healthy rats; and (2) to study the acute response to dietary protein rehabilitation, and protein synthesis through regulation of the mTORC1 signaling pathway and the polysome formation in the rat liver after a period of protein restricted diet. We found that the combination of BCr, SCr or S are the best proteins for dietary protein rehabilitation due to the significant increase in body weight, the activation of the mTORC1-signaling pathway in liver and muscle, and liver polysome formation.

## 2. Materials and Methods

### 2.1. Animal Protocol and Diets

#### 2.1.1. Study 1: Effect of Different Types of Vegetable Proteins on Healthy Rats

The purpose of this study was to determine the best protein or combination of proteins to ensure an optimal weight gain in healthy rats. Male Sprague-Dawley rats (3–4 weeks old, 180–200 g, *n* = 200) with free access to water were randomly assigned to one of eight experimental diets (*n* = 25 per group): (1) 20% casein (C) (control diet); (2) 20% soy protein (S); (3) 20% black bean protein (B); (4) 20% corn protein (Cr); (5) 20% pea protein (P); (6) 20% spirulina (Sp); (7) 20% sesame protein (Se); and (8) 10% B + 10% Cr (BCr). To synchronize food intake during the day, the rats were trained to consume one of the eight experimental diets during a restricted period of 10 h (08:00–18:00) for 21 days to guarantee similar food intake to animals fed ad libitum. The animals were weighed every other day, and food consumption was quantified every day. On Day 21, rats previously anesthetized with CO_2_ were euthanized by decapitation at fasting, 30 min, 60 min, 90 min and 120 min (*n* = 5 per time) after starting to eat their corresponding diets. All diets contained the same amount of nutrients with the exception of the type of protein that was adjusted to 20% taking into account the purity of the protein source as recommended by the American Institute of Nutrition [32]. The amino acid composition of each dietary protein is presented in Table S1. The composition of the experimental diets is presented in Table 1. The rat livers were rapidly excised, frozen in liquid nitrogen and stored at −70 °C. Blood samples were obtained after decapitation, and serum was separated and frozen at −20 °C to measure biochemical variables. The animal protocol was approved by the Institutional Animal Care and Research Advisory Committee of the Instituto Nacional de Ciencias Médicas y Nutrición SZ in Mexico City (CINVA 277).

#### 2.1.2. Study 2: Effect of Different Types of Vegetable Proteins on Protein Restricted Rats

The purpose of this study was to evaluate specific proteins or combination of proteins after a protein restricted period. Male Sprague-Dawley rats (3–4 weeks old, 180–200 g, *n* = 84) were fed a low-protein diet (LP; 0.5% casein) and allowed free access to water for 21 days. To synchronize food intake, the rats were trained to consume the experimental diet during a restricted period of 10 h (8:00–18:00). On Day 22, the rats were randomly assigned to one of the seven experimental diets (*n* = 12 per group): (1) Low protein (LP); (2) C; (3) S; (4) B; (5) Cr; (6) BCr; and (7) 10% S + 10% Cr (SCr). The composition of the experimental diets is presented in Table 1. On Days 1 and 7 of the dietary protein rehabilitation, rats previously anesthetized with CO_2_ were euthanized by decapitation (*n* = 6 per day) at 60 min after starting to eat their corresponding diets. The rat livers and muscles were rapidly excised, frozen in liquid nitrogen and stored at −70 °C. Blood samples were obtained after decapitation, and serum was separated and frozen at −20 °C to measure biochemical variables. The animal protocol was approved by the Institutional Animal Care and Research Advisory Committee of the Instituto Nacional de Ciencias Médicas y Nutrición S.Z in Mexico City (CINVA 277).

### 2.2. Biochemical Parameters

Blood was collected in tubes with gel and clot activator (BD Vacutainer, Franklin Lakes, NJ, USA) and centrifuged at 1000× *g* for 15 min to obtain serum. Serum glucose was measured using an YSI2700 Select Biochemistry Analyzer (YSI Incorporated, Yellow Sprig, OH, USA). Serum cholesterol and triglycerides were quantified by a Cholesterol FS* kit and Triglycerides FS* kit (DiaSys Diagnostic System GmbH, Holzheim, Germany). Insulin and glucagon were measured using a rat-specific radioimmunoassay kit (Millipore, St. Charles, MO, USA). Serum homocysteine (Hcy) was determined using Hcy kit (Axsym System Abbott, Chicago, IL, USA).

### 2.3. Determination of Plasma Amino Acids

The concentration of amino acids in the plasma was determined in triplicate by HPLC. A total of 150 µL of plasma was mixed with 38 µL of trichloroacetic acid, incubated for 30 min at 4 °C and centrifuged at 4 °C at 13,500 rpm for 12 min. The supernatant was used for derivatization of amino acids with OPA (*O*-Phthaldialdehyde) and FMOC (9-fluorenylmethylchloroformate). A sample was injected to an analytic column Agilent ZORBAX Eclipse AAA size 4.6 × 150 mm, 5 µm coupled to a fluorescence detector at 340 nm excitation and 450 nm emission (Agilent G1321B) for analysis. We used an HPLC Agilent 1260 Infinity system coupled to a binary pump (Agilent G1312B) and a robotic auto-sampler (Agilent G1367B).

### 2.4. Real-Time Quantitative PCR

Total RNA from livers was extracted according to the method of Chomczynski and Sacchi [33], followed by determination of integrity, concentration and purity. cDNA synthesis was carried out using an M-MLV reverse transcriptase enzyme and oligo (dT) 12–18 primer (Invitrogen, Carlsbad, CA, USA). The mRNA levels were measured by real-time quantitative PCR using Taqman Universal Master Mix (Applied Biosystems, Roche, Branchburg, NJ, USA). The PCR scheme used was 50 °C for 2 min, 95 °C for 10 min, and 40 cycles of 95 °C for 15 s followed by 60 °C for 1 min. TaqMan fluorogenic assays (Applied Biosystems, Foster City, CA, USA) and SYBR Green I^®^ (Sigma-Aldrich, St. Louis, MO, USA) were used according to the manufacturer’s instructions, using β-actin as the univariate control. The primers are shown in Table S2. The relative amounts of mRNA were calculated using the ∆∆*C*t method with an efficiency adjustment according to the Pfaffl equation [34].

### 2.5. Western Blot Analysis

Proteins were extracted using RIPA (Radioimmunoprecipitation Assay) lysis buffer (1% IGEPAL CA-630, 0.5% sodium deoxycholate, 0.1% sodium dodecyl sulfate, 1 mM sodium fluoride, 2 mM sodium orthovanadate and complete protease inhibitor cocktail tablets (Roche Applied Science, Mannheim, Germany), all dissolved in phosphate-buffered saline). The protein concentration was measured using the Lowry method with the DC Protein Assay (Bio-Rad., Hercules, CA, USA). Forty micrograms of total protein were heated for 5 min in Laemmli sample buffer (Bio-Rad, Hercules, CA, USA) and loaded on 8%–10% polyacrylamide gels, separated by sodium dodecyl sulfate polyacrylamide gel electrophoresis and transferred to polyvinylidene difluoride membrane. Blots were blocked for 1 h at room temperature with 5% blotting grade blocker nonfat dry milk (Bio-Rad, Hercules, CA, USA) and incubated overnight at 4 °C with the following primary antibodies: mTOR (cat #2972, dil 1:1000), pmTOR (Ser 2481) (cat #2974, dil 1:1000), p70S6K1 (Thr 389) (cat #9234, dil 1:1000), eIF4G (cat #2498, dil 1:1000) and GCN2 (cat #3302, dil 1:1000), eIF2α (cat#9722, dil 1:6000), peIF2α (Ser 51) (cat #9721, dil 1:500) were obtained from Cell Signaling Technology (Beverly, MA, USA). Antibodies against p70S6K1 (cat sc-230, dil 1:3000), SREBP-1 (cat sc-366, dil 1:250), FAS (cat sc-20140, dil 1:1000), SNAT2 (cat sc-67081, dil 1:800), β-actin (cat sc-1615, dil 1:1000) and tubulin (cat sc-7396, dil 1:1000) were obtained from Santa Cruz Biotechnology (Santa Cruz, CA, USA). Appropriate secondary antibodies were obtained from Santa Cruz Biotechnology (Santa Cruz, CA, USA). The blots were then incubated with anti-rabbit or -goat immunoglobulin G antibody conjugated with horseradish peroxidase (Santa Cruz Biotechnology, Santa Cruz, CA, USA) diluted 1:3500. The blots were developed by the enhanced chemiluminescence method with Immobilon Western Chemiluminescent HRP substrate (Millipore, Billerica, MA, USA) the blot image was acquired through the ChemiDoc MP imaging System (Bio-Rad, Hercules, CA, USA). The samples were analyzed three times in independent blots. Semiquantification of the bands was carried out by optical densitometry and analyzed using the ImageJ digital imaging processing software (ImageJ 1.48v, National Institutes of Health, Bethesda, MD, USA). The expression of each protein analyzed was normalized with γ-tubulin.

### 2.6. Polyribosome Studies

Rat frozen liver (400–500 mg) was homogenized in ice-cold Dounce homogenizer with 2 mL of lysis buffer (20 mM Tris HCl pH 7.4, 150 mM NaCl, 5 mM MgCl_2_, 1 mM DTT, 20 U/mL RNaseOUT (Invitrogen, Carlsbad, CA, USA) and protease inhibitor (Roche Applied Science, Mannheim, Germany)) and centrifuged at 11,000× *g* for 15 min at 4 °C. The supernatant was collected and diluted in lysis buffer supplemented with 1% NP-40 and 1% sodium deoxycholate and layered onto a 50% sucrose cushion (which will allow the sedimentation of heavy complexes such as polysomes but not light complexes such as 40S, 60S and 80S) in buffer A (20 mM Tris HCl pH 7.4, 100 mM NaCl, 3 mM MgCl_2_, 1 mM DTT, 5 U/mL RNaseOUT (Invitrogen, Carlsbad, CA, USA), and protease inhibitor (Roche Applied Science, Mannheim, Germany)) and centrifuged for 2 h at 200,000× *g* in a Beckman SW-40Ti rotor at 4 °C. The pellet was resuspended in 1 mL of lysis buffer supplemented with 1% NP-40 and 1% sodium deoxycholate and incubated on ice for 30 min. RNA was quantified, and 7.5 OD_260_ units were loaded onto the 15% to 55% sucrose gradient in buffer A (20 mM Tris HCl pH 7.4, 100 mM NaCl, 3 mM MgCl_2_, 1mM DTT, 5 U/mL RNaseOUT (Invitrogen, Carlsbad, CA, USA), supplemented with complete mini EDTA-free protease inhibitor cocktail tablets (Roche Applied Science, Mannheim, Germany) and centrifuged for 2 h 30 min at 230,000× *g* using a Beckman SW-40Ti rotor at 4 °C. Gradients were fractionated using an Automated Density Fractionation System (Teledyne Isco, Lincoln, NE, USA) with continuous monitoring of absorbance at 254 nm, the polyribosomal profile was edited on chart paper. The area under the curve of the profile was determined using the program ImageJ digital imaging processing software (ImageJ 1.48v).

### 2.7. Statistical Analysis

Values are expressed as the means ± SEM. The data were analyzed using GraphPad Prism (version 6.0f; Graph Pad Software, Inc., La Jolla, CA, USA). Differences were considered to be significant when *p* < 0.05. In Study 1, the final weight gain, food intake, serum biochemical parameters, gene and protein expression levels were analyzed by 1-factor ANOVA. Means were analyzed by Tukey’s post hoc analysis. In Study 2, the initial body weight, food intake, body weight d0, body weight d7, serum biochemical parameters, gene and protein expression levels were analyzed by 1-factor ANOVA. Means were analyzed by Tukey’s post hoc analysis.

## 3. Results

### 3.1. Study 1

#### 3.1.1. Weight Gain, Food Intake and Feed Conversion Ratio of Healthy Rats Fed Different Types of Proteins

The final body weight was significantly higher (*p* < 0.05) in the rats fed C, BCr, P, S and B than in the rats fed Sp, Se and Cr. The mean daily food intake was significantly higher (*p* < 0.05) in the rats fed C, B and Sp than in the rats fed S, BCr, P, Se and Cr (Table 2). The feed conversion ratio (FCR) that indicates the efficiency of the experimental diets to produce an optimal weight gain was significantly higher (*p* < 0.05) in the BCr and P groups, meanwhile rats fed Sp, Se or Cr showed the lowest FCR.

#### 3.1.2. Serum Biochemical Parameters and Homocysteine (Hcy) of Healthy Rats Fed Different Types of Proteins

**Figure 1:**
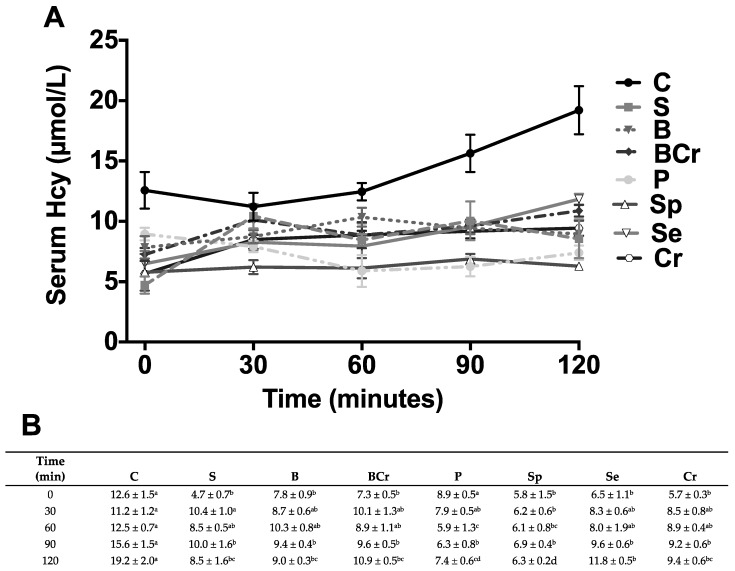
Serum Homocysteine (Hcy) concentration in healthy rats fed different types of dietary protein: (**A**) Fasting serum Hcy concentration and after 30, 60, 90 and 120 min of feeding casein (C), soy protein (S), black bean (B), black bean + corn (BCr), pea (P), spirulina (SP), sesame (Se) or corn (Cr); and (**B**) values are means ± SEM, *n* = 5. Different letter superscript indicates significant differences among rows, *p* < 0.05, a > b > c.

All serum biochemical parameters after 1 h refeeding in all groups were within the normal range. However, there was a significant difference in several parameters in some groups (Table 2). The more relevant differences were observed in the groups fed P, Se or Cr with the highest postprandial serum glucose concentrations (*p* < 0.05). The groups fed C or Se had the highest (*p* < 0.05) postprandial insulin concentrations. The highest glucagon concentration was observed in the B group (*p* < 0.05). The B group had the lowest insulin/glucagon ratio, whereas the C and Se groups had the highest insulin/glucagon ratio (*p* < 0.05). Since Sp contains nucleic acid and the biochemical degradation ends by producing uric acid, we determined serum uric acid concentration in rats fed Sp resulting in normal values (81.3 ± 6.0 µmol/L). Previous studies have demonstrated that the consumption of soy protein reduces serum Hcy concentration [35] due to that the limiting amino acid in soy is methionine, thus we assessed whether other different types of dietary vegetable proteins used in the present study regulates serum homocysteine concentration. Interestingly, rats fed with all different types of vegetable protein had significantly lower serum Hcy concentration at 90 and 120 min than the C group. Hcy concentration was significantly higher in fasting rats fed C with respect to all groups with exception of P group. The average fasting serum Hcy in rats fed vegetable protein was 6.83 ± 0.43 µmol/L, whereas, in the C group, it was 12.6 ± 1.5 µmol/L. After two hours of feeding, rats fed vegetable proteins, had average serum Hcy concentration of 8.9 ± 0.5, whereas the C group rats had 19.2 ± 2.0 µmol/L (Figure 1A), a value that is considered as hyperhomocysteinemia [36].

#### 3.1.3. Expression of the mTORC1-Signaling Pathway in the Livers of Healthy Rats Fed Different the Types of Proteins

**Figure 2:**
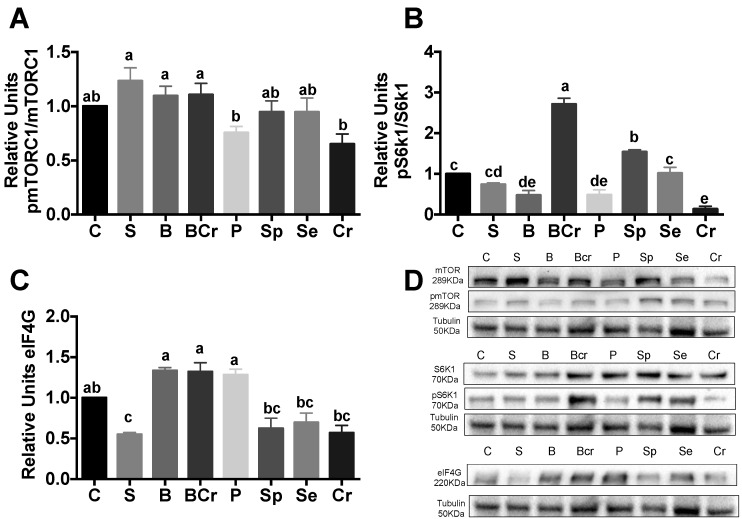
Western blot analysis and quantification of the: (**A**) phosphorylation of mTORC1; (**B**) phosphorylation of S6K1; (**C**) protein abundance of eIF4G in livers of healthy rats fed different types of dietary proteins and (**D**) representative immunoblot. Values are means ± SEM, *n* = 3. Different letter superscript indicates significant differences among groups, *p* < 0.05, a > b > c.

After 1 h of feeding, the p-mTORC1/mTORC1 ratio was significantly increased (*p* < 0.05) in the S, B and BCr groups, while the Cr and P groups had lower phosphorylation of mTORC1 (Figure 2A). The BCr group had the highest p-S6K1/S6K1 ratio among the experimental groups (*p* < 0.05) indicating a possible increase in ribosomal biogenesis [37]. Interestingly, the Cr group exhibited the lower phosphorylation of S6K1 (Figure 2B) indicating that, although Cr is rich in leucine that can stimulate mTORC1 [38], the deficiency of lysine and tryptophan in Cr could repress the activity of S6K1. We measured the expression of eIF4G, a translation initiation factor involved in the formation of eIF4E [39], and we observed that the expression of eIF4G was significantly increased (*p* < 0.05) in the B, BCr and P groups; meanwhile, the S, Cr, Sp and Se groups exhibited lower expression (*p* < 0.05) of eIF4G (Figure 2C).

#### 3.1.4. Expression of the Amino Acid Transporter SNAT2 in the Liver of Healthy Rats Fed Different Types of Proteins

**Figure 3:**
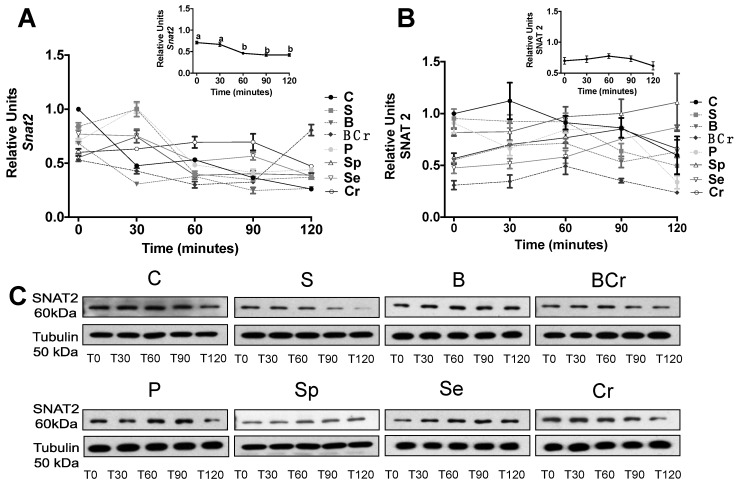
mRNA gene expression and Western blot analysis of SNAT2 in liver of healthy rats fed different types of dietary protein: (**A**) *Snat2* mRNA abundance; (**B**) protein abundance of *Snat2*; and (**C**) representative immunoblot of SNAT2. Values are means ± SEM, *n* = 3. Different letter superscript indicates significant differences among different times of feeding, *p* < 0.05, a > b > c.

Gene and protein expression of SNAT2 was not modified by the type of dietary protein (Figure 3A,B), however it was increased during the fasting period and decreased after feeding period (inset Figure 3A,B) (*p* < 0.05).

#### 3.1.5. Liver Gene and Protein Expression of the Transcription Factor SREBP1c and Its Target Enzyme FASN of Healthy Rats Fed Different Types of Proteins

**Figure 4:**
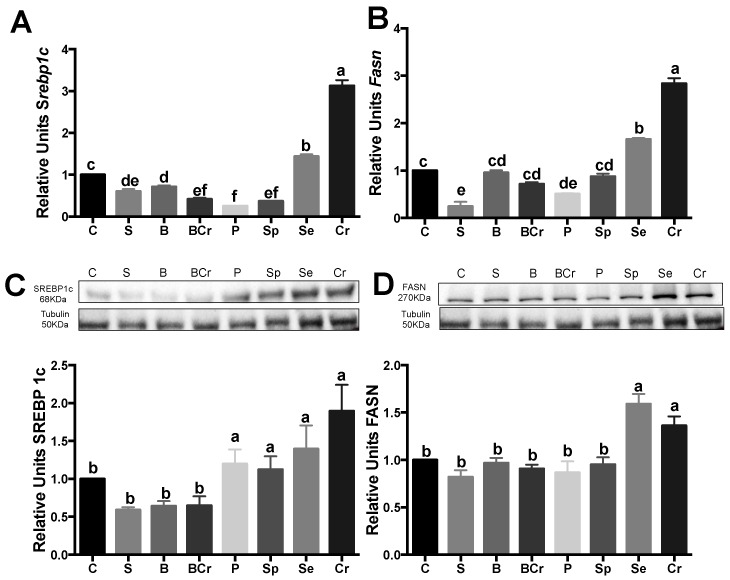
mRNA gene expression and Western blot analysis of SREBP1C and FASN in healthy rats: (**A**) *Srebp1c* and (**B**) *Fasn* mRNA abundance; (**C**) protein abundance of SREBP1c; and (**D**) FASN. Values are means ± SEM, *n* = 3. Different letter superscript in the bars indicates significant differences among groups, *p* < 0.05, a > b > c.

Diets low in lysine lead to fatty liver [40]. For this reason, we assessed the gene expression of the transcription factor *Srebp1c*, which is involved in fatty acid synthesis [41]. Srebp1c expression was significantly increased (*p* < 0.05) in the Cr and Se groups by 3.1- and 1.4-fold, respectively, with respect the C group. Interestingly, the S, BCr, Sp and P groups showed the lowest gene expression of Srebp1c (Figure 4A). The protein expression of SREBP1c was significantly increased (*p* < 0.05) in the rats fed P, Sp, Se and Cr, while the rats fed S, B and BCr showed the lowest expression of SREBP1C (Figure 4C). The fatty acid synthase gene (*Fasn*), the target gene of SREBP-1c, was significantly increased (*p* < 0.05) in the Cr group followed by the Se group after refeeding (Figure 4B). The protein expression of FASN increased in the Se and Cr groups after refeeding by 59% and 36.2%, respectively, in comparison with the C group (Figure 4D).

### 3.2. Study 2

#### 3.2.1. Weight Gain, Food Intake and Feed Conversion Ratio of Rats Fed Different Types of Proteins after a Protein Restricted Period

After the protein restricted period, the rats had 18% body weight loss. Subsequently, the rats underwent a dietary protein rehabilitation with different types and combinations of proteins. The rats fed LP for seven days lost an additional 4% of body weight, while the rats fed C, BCr or SCr had the highest (*p* < 0.05) final body weight gain after seven days of treatment. The group with the lowest (*p* < 0.05) final body weight gain was the Cr group. The mean daily intake was similar in all groups, with the exception of the rats fed Cr, who had similar food intake to those fed LP (Table 3). The highest FCR was seen in the C or S groups whereas the lowest FCR was seen in the SCr and Cr groups at Day 7.

#### 3.2.2. Serum Biochemical Variables of Rats Fed Different Types of Proteins after a Protein Restricted Period

The groups fed C or S had the highest (*p* < 0.05) serum glucose concentration, which were slightly higher than the normal value. The concentrations of serum triglycerides and cholesterol in all groups were within the normal range. Interestingly, the rats fed the C diet had the highest (*p* < 0.05) level of serum insulin, which was high enough to be considered hyperinsulinemia, whereas the rest of the groups had normal insulin concentrations. The groups with the highest (*p* < 0.05) glucagon concentrations were SCr and B. The B and LP groups had the lowest (*p* < 0.05) insulin/glucagon ratios, whereas the C group had the highest insulin/glucagon ratio (*p* < 0.05). We observed that rats fed C, S and B had the highest increase (*p* < 0.05) in albumin concentration, relative to Day 1, after seven days of eating the experimental diets. Interestingly, the rats fed Cr showed the lowest (*p* < 0.05) increase in albumin concentration (Table 3).

#### 3.2.3. Plasma Amino Acids of Rats Fed Different Types of Proteins after a Protein Restricted Period

Rats fed LP had the lowest total amount of indispensable plasma amino acids (IAA) and the lowest branched amino acid (BCCA) concentration among the experimental groups. Protein restricted rats fed any of the dietary proteins for rehabilitation showed a significant increase in the concentration of ΣIAA and ΣBCAA (*p* < 0.05). However, the C group showed the highest increase in ΣBCAA and ΣIAA. Interestingly, rats fed SCr exhibited a similar increase in ΣIAA concentration followed by Cr, S, B and BCr. Even though rats fed Cr had higher ΣIAA than S, the limiting amino acids in Cr were tryptophan and lysine (Table 4).

#### 3.2.4. Expression of the mTORC1-Signaling Pathway in the Livers of Rats Fed Different Types of Proteins after a Protein Restricted Period

**Figure 5:**
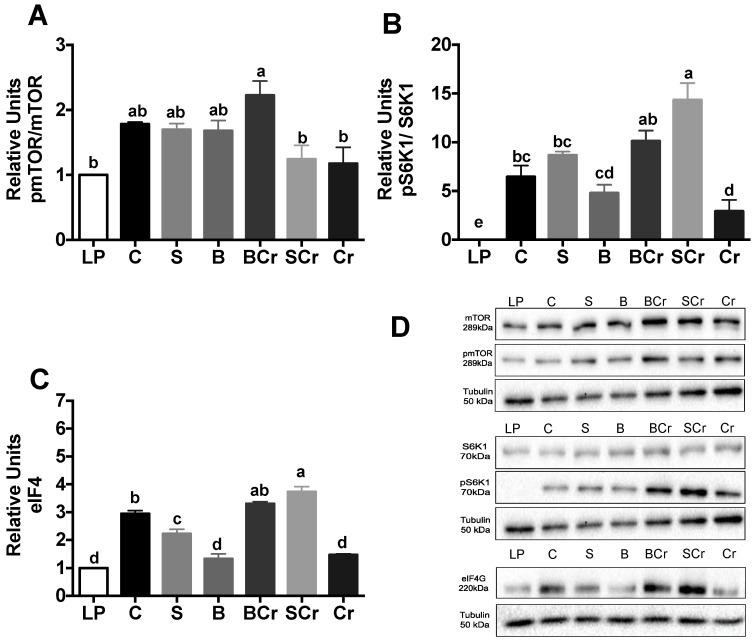
Western blot analysis and quantification of the: (**A**) phosphorylation of mTORC1; (**B**) phosphorylation of S6K1; and (**C**) protein abundance of eIF4G in the livers of rats fed different types of proteins after a protein restricted period; and (**D**) representative Western blot analysis. Values are means ± SEM, *n* = 3. Different letter superscript indicates significant differences among groups, *p* < 0.05, a > b > c.

The major differences in the mTORC1-signaling pathway in the liver were observed after one day of consuming the experimental diets. The BCr group showed the highest increase in the phosphorylation of mTORC1 (*p* < 0.05) (Figure 5A), whereas the SCr group showed the highest pS6K1/S6K ratio (*p* < 0.05) (Figure 5B), and the SCr group followed by the BCr group showed the highest expression of eIF4G (*p* < 0.05) (Figure 5C). These results suggest that the combination of dietary proteins significantly increases the hepatic mTORC1-signaling pathway, which could be related to an increase in protein synthesis.

#### 3.2.5. Expression of the Amino Acid Transporter SNAT2 in the Liver of Rats Fed Different Types of Proteins after a Protein Restricted Period

**Figure 6:**
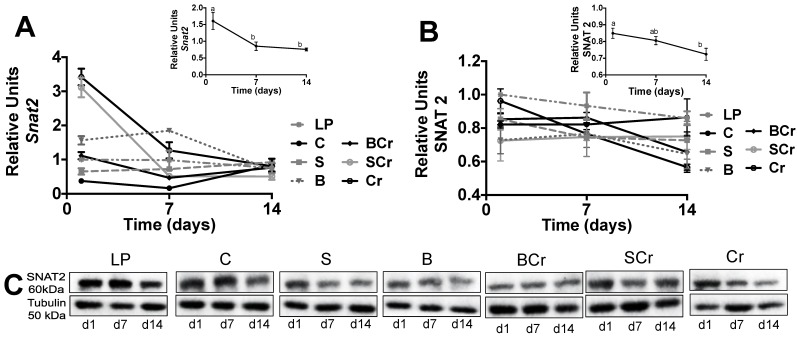
mRNA gene expression and Western blot analysis of SNAT2 in liver of rats fed different types of proteins after a protein restricted period: (**A**) *Snat2* mRNA abundance; (**B**) protein abundance of *Snat2*; and (**C**) representative Western blot analysis. Values are means ± SEM, *n* = 3. Different letter superscript indicates significant differences among time, *p* < 0.05, a > b > c.

Previous studies demonstrated that SNAT2 was upregulated in response to amino acid deprivation [42]. Thus, we evaluated SNAT2 as a possible amino acid sensor and if it could be regulated by the physiological state. The gene expression of *Snat2* was significantly increased (*p* < 0.05) in the SCr and Cr groups after one day of eating the experimental diets (Figure 6A); interestingly after seven and 14 days of eating the experimental diets there is a significant decrease (*p* < 0.05) in the gene expression of *Snat2* (Figure 6B) regardless the experimental diet indicating adaptive regulation, a process in which amino acid starvation induces the transport activity [43]. The protein expression of SNAT2 was significantly elevated (*p* < 0.05) after one day of eating the experimental diets and decreased after seven and 14 days of dietary protein rehabilitation (Figure 6C).

#### 3.2.6. Expression of GCN2 and eIF2α in the Liver of Rats Fed Different Types of Proteins after a Restricted Protein Period

**Figure 7:**
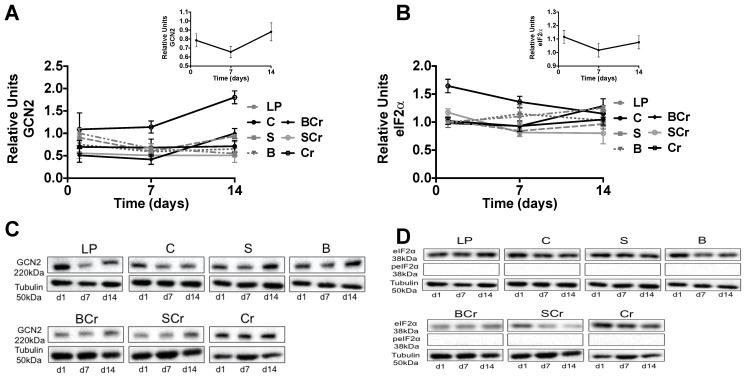
Western blot analysis and quantification of GCN2 and eIF2α in liver of rats fed different types of proteins after a protein restricted period: (**A**) representative Western blot analysis of GCN2 and (**B**) eIF2α; (**C**) protein abundance of GCN2; and (**D**) protein abundance of eIF2α. Values are means ± SEM, *n* = 3, *p* < 0.05, a > b > c.

Rats fed Cr showed the highest expression of GCN2 after seven and 14 days (*p* < 0.0001) of eating the experimental diets, while rats fed C, S, B, BCr or SCr showed the lowest expression of GCN2 (Figure 7A,C). Despite the abundance of GCN2 in all groups, we did not observed phosphorylation of its target protein eIF2α (Figure 7B,D).

#### 3.2.7. Liver Gene and Protein Expression of the Transcription Factor SREBP1c and Its Target Enzyme FASN of Rats Fed Different Types of Proteins after a Protein Restricted Period

**Figure 8:**
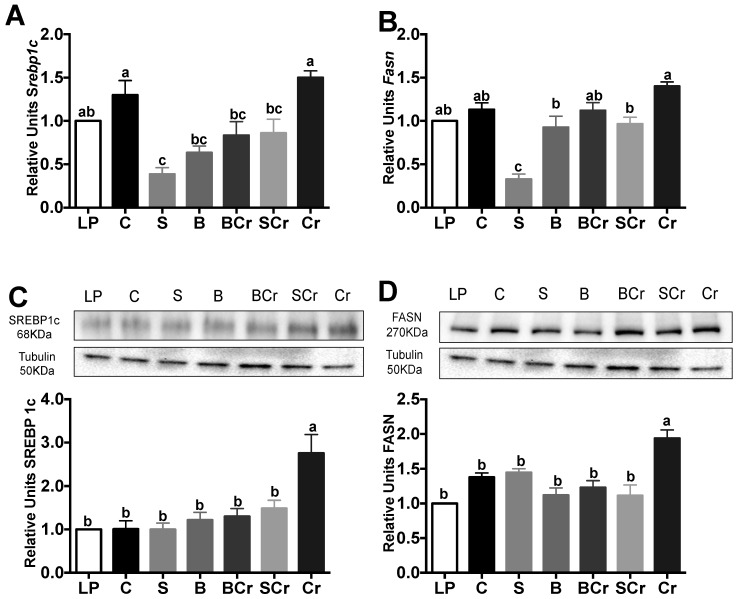
mRNA gene expression and Western blot analysis of SREBP1C and FASN in rats fed different types of proteins after a protein restricted period: (**A**) *Srebp1c* mRNA abundance; (**B**) *Fasn* mRNA abundance; (**C**) abundance of SREBP1c; and (**D**) abundance of FASN. Values are means ± SEM, *n* = 3. Different letter superscript indicates significant differences among groups, *p* < 0.05, a > b > c.

*Srebp1c* gene expression was significantly increased in the C, Cr and LP groups (*p* < 0.05), and, interestingly, the S group showed the lowest expression of *Srebp1c* (*p* < 0.05) (Figure 8A). SREBP1c protein expression was significantly increased (*p* < 0.05) in the Cr group by 2.7-fold in comparison with the LP group (Figure 8C). The gene expression of *Fasn* was significantly increased (*p* < 0.05) in the Cr group, and the rats fed S had the lowest expression of *Fasn* (*p* < 0.05) (Figure 8B). The highest protein expression of FASN was observed in the Cr group and was significantly decreased by the combination of Cr with S or B (Figure 8D).

#### 3.2.8. Hepatic Polysome Profile of Rats Fed Different Types of Proteins after a Protein Restricted Period

**Figure 9:**
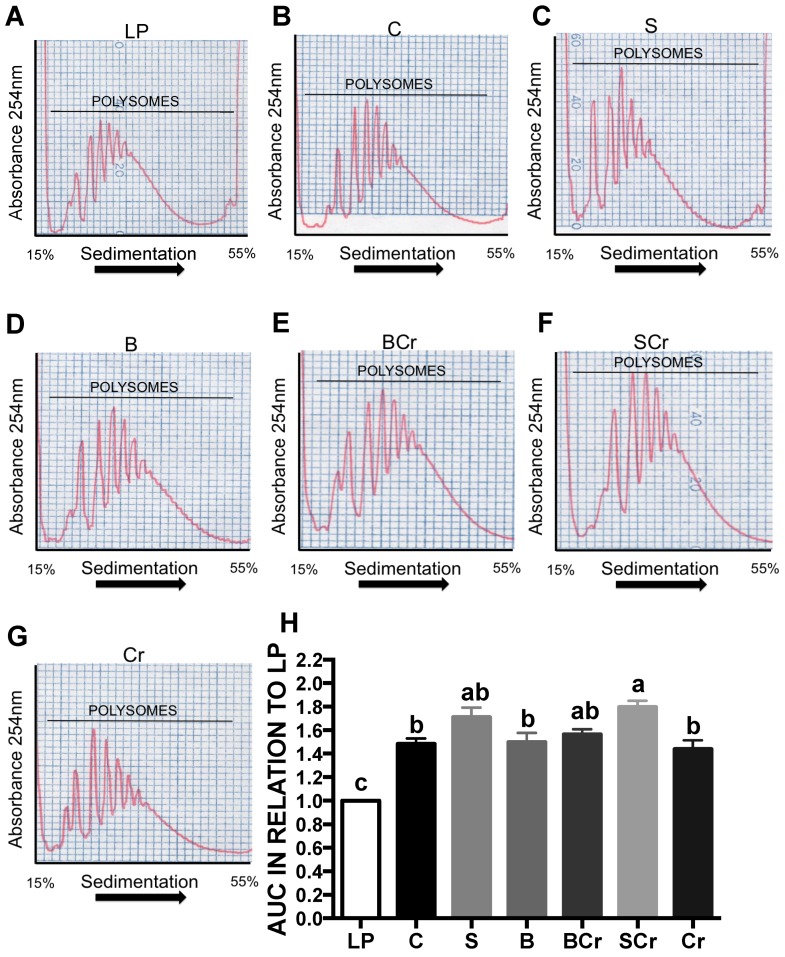
Hepatic polysome profile of rats fed different types of dietary proteins after a protein restricted period: (**A**) 0.5% casein; (**B**) casein; (**C**) soy; (**D**) black bean; (**E**) black bean + corn; (**F**) soy + corn; (**G**) corn; and (**H**) area under the curve analysis. The values are mean ± SEM, *n* = 3 per group. Different letter superscript indicates significant differences among groups, *p* < 0.05, a > b > c.

The results indicate that each type of dietary protein generates a specific pattern of expression and phosphorylation of factors involved in the initiation of translation leading to a specific pattern of polysome formation. In general, there was a significant increase (*p* < 0.05) in polysome formation in all groups in comparison with the LP group (Figure 9A–G). The polysome profiles in the livers of rats fed SCr, S and BCr showed the highest polysome formation (Figure 9H) indicating a possible increase in the initiation of protein translation.

#### 3.2.9. Expression of the mTORC1-Signaling Pathway in the Muscles of Rats Fed Different Types of Proteins after a Protein Restricted Period

**Figure 10:**
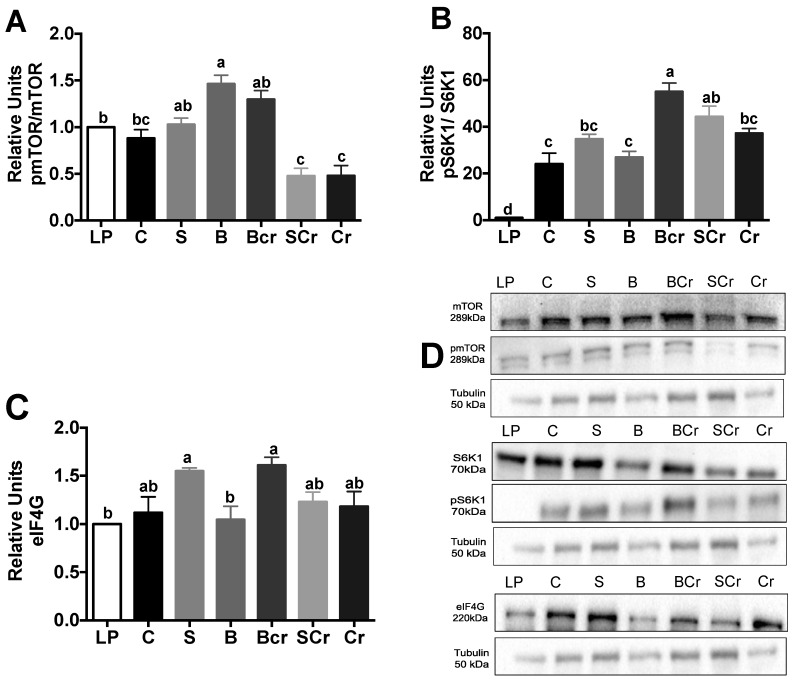
Western blot analysis and quantification of the: (**A**) phosphorylation of mTORC1; (**B**) phosphorylation of S6K1; (**C**) protein abundance of muscle eIF4G; and (**D**) representative Western blot of rats fed different types of protein for seven days after a protein restricted period. Values are means ± SEM, *n* = 3. Different letter superscript indicates significant differences among groups, *p* < 0.05, a> b > c.

Skeletal muscle is one of the main sites of protein synthesis. After a meal, as nutrients are absorbed, the rate of protein synthesis increases [44]. Because the Cr diet contains three times the leucine requirement, and leucine increases protein synthesis, we were interested in evaluating whether the Cr diet could modify muscle mTORC1-signaling pathway. No changes were observed in the phosphorylation of mTORC1 and S6K1 in muscle after one day of consuming the different experimental diets (Figure S1). Interestingly, after seven days of consuming the experimental diets, we observed a significant increase in the mTORC1 phosphorylation in the B and BCr groups followed by the S group. The Cr and SCr groups showed the lowest p-mTORC1/mTORC1 ratio (Figure 10A). The BCr group followed by the SCr group showed the highest (*p* < 0.05) p-S6K1/S6k1 ratio among the groups (Figure 10B). The protein expression of eIF4G in muscle was significantly increased in the S and BCr groups (Figure 10C).

## 4. Discussion

Undernutrition and malnutrition are serious health problems that affect 795 million individuals worldwide. The use of dietary proteins, particularly the combination of legumes and cereals, has been reported to be a good strategy for combating undernutrition [8]. Legume proteins are a good source of lysine but are deficient in methionine, and cereals are rich in methionine but deficient in lysine and tryptophan. Thus, the combination of these two proteins results in proteins with a quality similar to those from animal sources [9,10]. Using nutrigenomics, it is currently possible to understand the mechanism of action at the molecular level that determines how these proteins or combinations of proteins can regulate protein synthesis through the activation of the mTORC1 signaling pathway after a protein restricted period. In order to know more about the possible sensor of amino acids in the cells, we also studied the transport system SNAT2. Certain amino acid transporters such as SNAT2 may have dual receptor-transporter function operating as transceptors [45]. Consequently, the activity of this amino acid transporter could potentially activate mTORC1 [24].

We assessed the effect of quality of different types of proteins. The quality of a protein is evaluated by expressing the content of the first limiting essential amino acid of the test protein as a percentage of the content of the same amino acid in a reference pattern of essential amino acids to identify the best proteins to use in the dietary protein rehabilitation experiment. Several studies have demonstrated the benefits of soy protein [46] on optimal animal growth, however less is known about the effects of other vegetable proteins. We found that C, S, P, BCr and B were the best proteins for adequate weight gain, and BCr and P proteins had the highest FCR, however, P was not included in the second part because the consumption of this protein produced high postprandial glucose concentration. The proteins with the lowest chemical scores were Sp (47), Se (42) and Cr (34), as was reflected by lower weight gain in these groups with respect to rest of the groups. Interestingly, rats fed vegetable proteins showed lower serum Hcy concentration than casein, indicating that consumption of vegetable protein could reduce the risk of atherosclerosis [47,48]. In the second part of the study, we included the combination of BCr and SCr since corn contains three times the leucine requirement and could have an additive effect on the activation of the mTORC1-signaling pathway and polysome formation. BCr was included in the first study since both proteins constitutes the staple diet for most Mexicans, and after analyzing the results of the first study we realize the importance of combination of proteins mainly a cereal with a legume as previously described [49] for this reason we included SCr combination.

The mTORC1 signaling pathway is highly regulated by insulin and amino acids [23]. To activate mTORC1, sufficient levels of amino acids are necessary and, in particular, the concentrations of leucine and arginine are critical [50]. On the other hand, the System A transporter SNAT2 besides its transporter activity, it has been involved in the activation of the mTORC1-signaling pathway [24]. Interestingly we observed that the expression of this transporter was increased in fasting and decreased after feeding the experimental diets regardless the type or quality of the dietary protein. These results indicate that the regulation of mTORC1 activity is independent of the SNAT2 activity. Previous studies demonstrated that SNAT2 was upregulated in response to amino acid deprivation indicating adaptive regulation, a process in which amino acid starvation induces the transport activity. Interestingly in the present study, we observed that SNAT2 was significantly increased during the fasting and decreased after refeeding independently of the type of protein indicating adaptive regulation. Despite the fact that the Cr diet had almost triple the amount of leucine recommended by the FAO, healthy rats fed the Cr diet showed a significant decrease in the phosphorylation of S6K1 probably due to the limiting amino acids Trp and Lys in this diet. Interestingly, when the Cr protein was combined with B in similar proportions, the phosphorylation of S6K1 was enhanced, indicating that complementation of these proteins could increase ribosomal biogenesis. Interestingly, low quality proteins such as Se or Cr stimulated SREBP1c and its target gene Fasn. This could be due to the low content of lysine. A previous report demonstrated that diets low in lysine cause impairment of growth and development of fatty liver [40]. In the present study, diets with Se or Cr exhibited the lowest lysine content, and this was associated with higher expression of lipogenic genes. However, when Cr is combined with B, the expression of these genes is significantly decreased. These results indicate that the quality of dietary proteins plays an important role in the regulation of fatty acid synthesis.

During the dietary protein rehabilitation after the protein restricted period, the combination of S or B with Cr increased the body weight of rats to a similar extent as that of control rats fed the C diet. This could be explained by an improvement in plasma ΣIAA mainly in the SCr group. It is important to point out that an increase in ΣIAA does not necessarily indicate a very good quality protein. The ΣIAA in the Cr group is similar to the C group, however the limiting amino acids in Cr group were Lys and Trp than can limit the protein synthesis through a decrease of the mTORC1-signaling pathway. The limiting amino acids play an important role in weight gain and the activation of the mTORC1-signaling pathway as seen in the Cr group. SCr or BCr are good protein combinations because they did not increase the serum insulin concentration as observed in the C group, indicating that complementation of dietary proteins could be a good strategy for dietary protein rehabilitation to avoid the development of hyperinsulinemia and lipogenesis. Interestingly, rats fed Cr showed an increase in the abundance of GCN2, probably due to the limitation of lysine and tryptophan inhibiting polysome formation and the ability to activate mTORC1-signaling pathway. On the other hand, the combination of SCr or BCr showed a similar pattern of activation of mTORC1, S6K1 and eIF4G increasing polysome formation that could indicate an increase in the initiation of protein synthesis in the liver after a protein restricted period.

During dietary protein rehabilitation, we observed changes in the mTORC1-signaling pathway at Day 1 in liver, whereas we observed changes until Day 7 in muscle. We could not compare the results between liver and muscle at Day 1 because most of the proteins involved in the mTORC1-signaling pathways were not observed in the Western blot analysis for muscle. The most relevant results indicate that the combination of SCr or BCr are the more adequate protein to regulate some proteins involved in mTORC1-signaling pathway such as S6K1, and eIF4G. Meanwhile, in muscle at Day 7, we observed an increase in p-S6K1 and eIF4G. These results indicate that the protein synthesis rate is faster in liver than in muscle, and it has been reported to be 77%/day in liver and 12%/day in muscle [51,52]. The activation of the mTORC1-signaling pathway in muscle is enhanced by amino acids, particularly leucine [23]; however, we observed that rats fed Cr, with the highest leucine content showed the lowest activation of the mTORC1-signaling pathway. This could be related to the lack of lysine and tryptophan in this protein. Interestingly, when Cr is combined with B or S, the quality of the dietary protein significantly increases and is able to sustain mTORC1 activation in liver and muscle. Differences in the activation of mTORC1 signaling pathway by the different dietary proteins could be due to content and the amino acid pattern of the dietary proteins.

## 5. Conclusions

Our results provide evidence that the optimal combination of some vegetable proteins complement the deficiency in amino acids of each source and improve the quality of the dietary protein similar to that of animal proteins. It is unrealistic to presume that a perfect diet can be formulated, one that can satisfy all circumstances [33], however mixing adequate vegetable proteins such as SCr, BCr or S could minimize cost while maintaining adequate growth, activate mTORC1-signaling pathway and increase eIF4G and liver polysome formation after a period of protein restricted diet. The results of the present study could be helpful in the development of local food supplements for the treatment of individuals with malnutrition, although further research is necessary to study other combinations of proteins.

## Figures and Tables

**Table 1:** Composition of experimental diets used in healthy and protein restricted rats.

Ingredients	C	LP	S	B	P	Sp	Se	Cr	BCr	SCr
Casein ^1^, g	219.8	5.5	0	0	0	0	0	0	0	0
Soy protein isolate ^2^, g	0	0	220	0	0	0	0	0	0	110.2
Black bean protein concentrate ^3^, g	0	0	0	287.5	0	0	0	0	144.3	0
Pea protein isolate ^4^, g	0	0	0	0	275.3	0	0	0	0	0
Spirulina ^5^, g	0	0	0	0	0	410.3	0	0	0	0
Sesame protein isolate ^6^, g	0	0	0	0	0	0	232	0	0	0
Corn protein isolate ^7^, g	0	0	0	0	0	0	0	336.2	167.4	167.9
Corn starch, g	390.4	520.4	389.9	371.7	373	331.8	386.6	352.6	362.2	371.3
Dextrinized cornstarch, g	126	172.7	127	110.2	111.4	69.7	126.2	89	99.6	108
Sucrose, g	94.2	130.8	95.3	78.7	79.8	38.1	94.8	57.2	68	76.3
Soybean oil, g	69.5	70	68.2	55.8	61.9	39.9	62.6	66.1	61	67.2
α-Cellulose, g	49.8	50	49.5	46.4	48.9	47.7	48.2	48.8	47.6	49.2
AIN-93-MX, g	35	35	35	35	35	35	35	35	35	35
AIN-93-VX, g	10	10	10	10	10	10	10	10	10	10
l-Cystine, g	3	3	3	3	3	3	3	3	3	3
Choline bitartrate, g	2.5	2.5	2.5	2.5	2.5	2.5	2.5	2.5	2.5	2.5
TBHQ, g	0.014	0.014	0.014	0.014	0.014	0.014	0.014	0.014	0.014	0.014
Total amount, g	1000	1000	1000	1000	1000	1000	1000	1000	1000	1000
Total protein content, % kcal	19.3	0.5	19.3	19.3	19.3	19.3	19.3	19.3	19.3	19.3
Total carbohydrate content, % kcal	65.5	84.3	65.5	65.5	65.5	65.5	65.5	65.5	65.5	65.5
Total fat content, % kcal	15.2	15.2	15.2	15.2	15.2	15.2	15.2	15.2	15.2	15.2
Energy density, kcal/g	4.1	4.1	4.1	4.1	4.1	4.1	4.1	4.1	4.1	4.1

^1^ Harlan Teklad; ^2^ SUPRO 710; ^3^ Black bean protein; ^4^ Nutralys pea protein, Roquette; ^5^ AEH, Solarium Biotechnology; ^6^ SESAPROT, Dipasa México; ^7^ INGREDION, México; TBHQ, tert-butylhydroquinone. C: casein; LP: low protein; S: soy protein; B: black bean protein; P: pea protein; SP: spirulina; SE: sesame protein; Cr: corn protein; BCr: black bean protein + corn protein; SCr: Soy protein + corn protein.

**Table 2:** Biochemical, anthropometric variables and feed conversion ratio of healthy Sprague-Dawley rats after one hour of feeding with different types of protein during 21 days.

	C	S	B	BCr	P	Sp	Se	Cr	*P* ^1^	*P* ^2^
Initial body weight, g	200.2 ± 1.3 ^b^	193.5 ± 2.5 ^bc^	195.5 ± 1.5 ^b^	197.1 ± 2.1 ^b^	186.7 ± 1.6 ^c^	199.3 ± 1.9 ^b^	199.8 ± 2.1 ^b^	212.5 ± 1.9 ^a^	0.001	0.001
Final body weight, g	255.4 ± 1.9 ^a^	239.8 ± 3.3 ^bc^	236.7 ± 2.3 ^bd^	248.9 ± 2.2 ^ac^	245.0 ± 3.0 ^ac^	228.4 ± 2.7 ^d^	225.9 ± 2.0 ^d^	229.6 ± 2.4 ^d^	0.001	0.001
Weight gain, day 4–21, g	55.2 ± 1.9 ^ab^	46.3 ± 2.3 ^b^	41.2 ± 2.2 ^c^	51.8 ± 2.3 ^bc^	58.3 ± 2.6 ^a^	29.2 ± 2.9 ^d^	26.1 ± 2.0 ^de^	17.1 ± 1.6 ^de^		
Food intake, g/day	19.0 ± 0.9 ^a^	16.8 ± 0.8 ^ab^	17.4 ± 0.4 ^a^	15.5 ± 0.5 ^b^	15.4 ± 1.0 ^b^	16.1 ± 0.7 ^a^	14.2 ± 0.4 ^b^	15.8 ± 0.5 ^b^		
Feed conversion ratio (FCR)	2.9 ± 0.1 ^bc^	2.7 ± 0.2 ^c^	2.4 ± 0.1 ^cd^	3.4 ± 0.2 ^ab^	3.9 ± 0.2 ^a^	1.8 ± 0.2 ^d^	1.8± 0.2 ^d^	1.1 ± 0.1 ^e^		
Serum										
Glucose, mmol/L	7.2 ± 0.3 ^ab^	6.8 ± 0.2 ^b^	6.2 ± 0.2 ^b^	7.0 ± 0.1 ^ab^	7.9 ± 0.3 ^a^	6.1 ± 0.4 ^b^	7.4 ± 0.2 ^a^	7.5 ± 0.2 ^a^		
Triglycerides, mmol/L	0.28 ± 0.1 ^b^	0.51 ± 0.1 ^ab^	0.5 ± 0.1 ^ab^	0.61 ± 0.1 ^ab^	0.58 ± 0.1 ^ab^	0.71 ± 0.1 ^a^	0.40 ± 0.1 ^ab^	0.63 ± 0.1 ^ab^		
Cholesterol, mmol/L	1.64 ± 0.50 ^b^	1.60 ± 0.06 ^b^	1.54 ± 0.05 ^b^	1.77 ± 0.12 ^b^	1.58 ± 0.07 ^b^	1.79 ± 0.07 ^a^	2.11 ± 0.10 ^a^	2.14 ± 0.06 ^a^		
Insulin, pmol/L	269.9 ± 5.2 ^a^	158.7 ± 3.7 ^cd^	55.8 ± 5.6 ^e^	160.5 ± 5.8 ^c^	167.0 ± 5.7 ^c^	168.4 ± 6.3 ^c^	204.7 ± 7.3 ^b^	134.0 ± 3.5 ^d^		
Glucagon, ng/L	75.3 ± 2.3 ^bc^	70.1 ± 1.1 ^bc^	103.3 ± 2.4 ^a^	70.3 ± 3.5 ^bc^	74.6 ± 3.3 ^bc^	65.2 ± 5.3 ^c^	44.9 ± 2.9 ^d^	86.4 ± 5.8 ^b^		
Insulin/Glucagon	3.8 ± 0.3 ^a^	2.3 ± 0.1 ^bc^	0.6 ± 0.1 ^d^	2.2 ± 0.1 ^bc^	2.3 ± 0.1 ^bc^	2.7 ± 0.3 ^b^	4.6 ± 0.3 ^a^	1.5 ± 0.1 ^cd^		

C, casein; S, soy protein; B, black bean protein; P, pea protein; Sp, spirulin protein; Se, sesame protein; Cr, corn protein. The values are mean ± SEM. Means in a row without a common letter differ, *p* < 0.05. Differences are based on one-way ANOVA. Tukey test was used as post hoc analysis; *P* ^1^, weight; *P* ^2^, weight × diet.

**Table 3:** Biochemical, anthropometric variables and feed conversion ratio of Sprague-Dawley rats fed different types of protein after a protein-restricted period.

		LP	C	S	B	BCr	SCr	Cr	*P* ^1^	*P* ^2^
Initial body weight, g		147.3 ± 2.0 ^a^	145.3 ± 1.6 ^abc^	144.6 ± 1.9 ^abc^	151.7 ± 1.6 ^a^	140.9 ± 1.5 ^bc^	137.5 ± 1.8 ^c^	143.2 ± 2.1 ^bc^	0.048	0.001
Body weight, day 7, g		114.7 ± 1.5 ^d^	157.7 ± 1.9 ^a^	138.6 ± 2.6 ^bc^	140.3 ± 2.6 ^bc^	154.7 ± 4.5 ^a^	149.2 ± 2.5 ^ab^	133.4 ± 2.1 ^c^	0.048	0.001
Food intake, g/day		8.39 ± 0.6 ^b^	18.22 ± 1.3 ^a^	15.82 ± 0.9 ^a^	14.44 ± 0.9 ^a^	17.61 ± 0.9 ^a^	17.96 ± 1.5 ^a^	10.64 ± 0.6 ^b^		
Feed conversion ratio, day 7		−3.7 ± 0.4 ^d^	0.8 ± 0.2 ^a^	0.9 ± 0.2 ^a^	−0.3 ± 0.1 ^bc^	−0.1 ± 0.1 ^b^	−1.1 ± 0.2 ^c^	−1.0 ± 0.1 ^d^		
Biochemical parameters	Day									
Glucose, mmol/L	7	8.5 ± 0.5 ^ab^	9.3 ± 0.2 ^a^	8.6 ± 0.5 ^ab^	7.8 ± 0.2 ^bc^	7.4 ± 0.2 ^bc^	7.0 ± 0.3 ^c^	7.7 ± 0.2 ^bc^		
Triglycerides, mmol/L	7	0.77 ± 0.1 ^b^	0.58 ± 0.2 ^b^	1.28 ± 0.1 ^a^	0.72 ± 0.1 ^b^	1.15 ± 0.1 ^a^	0.92 ± 0.1 ^b^	0.85 ± 0.1 ^b^		
Cholesterol, mmol/L	7	1.66 ± 0.1 ^b^	2.73 ± 0.1 ^a^	2.71 ± 0.1 ^a^	1.96 ± 0.1 ^b^	2.38 ± 0.2 ^a^	2.39 ± 0.2 ^a^	2.52 ± 0.1 ^a^		
Insulin, pmol/L	7	83.5 ± 6.8 ^d^	445.0 ± 15.3 ^a^	218.5 ± 16.5 ^b^	148.1 ± 10.9 ^c^	230.3 ± 12.9 ^b^	249.5 ± 16.9 ^b^	239.0 ± 13.8 ^b^		
Glucagon, ng/L	7	74.3 ± 3.7 ^c^	88.9 ± 6.3 ^bc^	99.0 ± 4.7 ^ab^	110.2 ± 2.2 ^a^	76.3 ± 3.2 ^bc^	114.1 ± 3.8 ^a^	105.2 ± 3.8 ^ab^		
Insulin/Glucagon	7	1.1 ± 0.1 ^c^	5.1 ± 0.4 ^a^	2.3 ± 0.3 ^bc^	1.4 ± 0.1 ^c^	3.0 ± 0.2 ^b^	2.2 ± 0.2 ^bc^	2.3 ± 0.1 ^bc^		
Albumin g/dL	Δ7-1	−0.25 ± 0.07 ^d^	1.60 ± 0.11 ^a^	1.32 ± 0.15 ^ab^	1.05 ± 0.24 ^ab^	0.73 ± 0.12 ^b^	0.66 ± 0.23 ^b^	0.30 ± 0.16 ^c^		

LP, low protein; C, casein; S, soy protein; B, black bean protein; Cr, corn protein. The values are mean ± SEM, intake. Means in a row without a common letter differ, *p* < 0.05. Differences are based on one-way ANOVA. Tukey correction was used as post hoc analysis. *P* ^1^, weight; *P* ^2^, weight × diet.

**Table 4:** Plasma amino acid concentration (µmol/mL) of Sprague-Dawley rats fed different types of protein after a protein restricted period.

AMINO ACIDS (µmol/mL)	LP	C	S	B	BCr	SCr	Cr
Asp	0.045 ± 0.001 ^c^	0.078 ± 0.003 ^a^	0.061 ± 0.002 ^b^	0.057 ± 0.003 ^bc^	0.055 ± 0.004 ^bc^	0.059 ± 0.002 ^bc^	0.056 ± 0.03 ^bc^
Glu	0.24 ±0.008 ^b^	0.26 ±0.006 ^a^	0.24 ± 0.005 ^b^	0.23 ± 0.001 ^b^	0.17± 0.003 ^c^	0.23 ± 0.006 ^b^	0.18 ± 0.004 ^c^
Ser	0.56 ± 0.01 ^c^	0.92 ± 0.03 ^ab^	1.08 ± 0.06 ^a^	0.88 ± 0.02 ^b^	0.83 ± 0.02 ^b^	0.97 ± 0.03 ^ab^	0.91 ± 0.05 ^ab^
His	0.055 ± 0.001 ^b^	0.100 ± 0.006 ^a^	0.095 ± 0.004 ^a^	0.079 ± 0.004 ^ab^	0.060 ± 0.005 ^b^	0.102 ± 0.008 ^a^	0.063 ± 0.006 ^b^
Gly	0.76 ± 0.01 ^c^	0.74 ± 0.02 ^c^	0.94 ± 0.02 ^b^	1.08 ± 0.02 ^a^	0.66 ± 0.02 ^dc^	0.65 ± 0.02 ^dc^	0.56 ± 0.02 ^d^
Thr	0.19 ± 0.03 ^b^	0.27 ± 0.05 ^ab^	0.37 ± 0.06 ^ab^	0.37 ± 0.02 ^ab^	0.31 ± 0.07 ^ab^	0.41 ± 0.05 ^a^	0.46 ± 0.02 ^a^
Arg	0.02 ± 0.002 ^c^	0.19 ± 0.001 ^a^	0.12 ± 0.016 ^b^	0.15 ± 0.011 ^ab^	0.14 ± 0.012 ^b^	0.21 ± 0.004 ^a^	0.16 ± 0.015 ^ab^
Ala	1.53 ± 0.03 ^cd^	1.68 ± 0.05 ^bc^	2.13 ± 0.06 ^a^	1.78 ± 0.04 ^b^	1.34 ± 0.05 ^d^	1.50 ± 0.07 ^cd^	1.39 ± 0.02 ^d^
Val	0.070 ± 0.006 ^d^	0.324 ± 0.016 ^a^	0.271 ± 0.009 ^ab^	0.242 ± 0.012 ^bc^	0.205 ± 0.008 ^c^	0.237 ± 0.013 ^bc^	0.215 ± 0.009 ^c^
Met	0.11 ± 0.009 ^ab^	0.15 ± 0.010 ^a^	0.11 ± 0.007 ^ab^	0.10 ± 0.007 ^b^	0.10 ± 0.005 ^b^	0.12 ± 0.009 ^ab^	0.12 ± 0.010 ^ab^
Cys	0.607 ± 0.004 ^c^	0.695 ± 0.013 ^b^	0.891 ± 0.027 ^a^	0.768 ± 0.016 ^b^	0.528 ± 0.008 ^de^	0.575 ± 0.012 ^d^	0.471 ± 0.022 ^e^
Phe	0.043 ± 0.004 ^b^	0.111 ± 0.009 ^a^	0.111 ± 0.005 ^a^	0.109 ± 0.008 ^a^	0.108 ± 0.003 ^a^	0.113 ± 0.006 ^a^	0.119 ± 0.006 ^a^
Tyr	0.040 ± 0.002 ^d^	0.185 ± 0.005 ^ab^	0.170 ± 0.008 ^ab^	0.140 ± 0.008 ^c^	0.145 ± 0.005 ^bc^	0.190 ± 0.015 ^a^	0.191 ± 0.003 ^a^
Ile	0.046 ± 0.002 ^d^	0.118 ± 0.004 ^a^	0.123 ± 0.04 ^a^	0.096 ± 0.002 ^b^	0.072 ± 0.03 ^c^	0.083 ± 0.05 ^bc^	0.066 ± 0.004 ^c^
Leu	0.060 ± 0.005 ^d^	0.201 ± 0.006 ^b^	0.179 ± 0.008 ^bc^	0.156 ± 0.003 ^c^	0.201 ± 0.011 ^b^	0.230 ± 0.011 ^a^	0.259 ± 0.012 ^a^
Lys	0.30 ± 0.006 ^cd^	0.49 ± 0.012 ^ab^	0.51 ± 0.022 ^a^	0.42 ± 0.012 ^b^	0.28 ± 0.016 ^d^	0.35 ± 0.012 ^bc^	0.17 ± 0.014 ^e^
Pro	0.31 ± 0.01 ^c^	0.68 ± 0.02 ^a^	0.48 ± 0.001 ^b^	0.34 ± 0.02 ^c^	0.45 ± 0.01 ^b^	0.39 ± 0.02 ^c^	0.51 ± 0.001 ^b^
Trp	0.036 ± 0.003 ^b^	0.093 ± 0.008 ^a^	0.078 ± 0.004 ^a^	0.031 ± 0.002 ^b^	0.024 ± 0.003 ^b^	0.037 ± 0.005 ^b^	0.018 ± 0.003 ^c^
Asn	0.032 ± 0.001 ^c^	0.065 ± 0.03 ^b^	0.116 ± 0.007 ^a^	0.70 ± 0.004 ^b^	0.75 ± 0.005 ^b^	0.108 ± 0.03 ^a^	0.081 ± 0.001 ^b^
Gln	0.81 ± 0.01 ^c^	0.89 ± 0.001 ^a^	0.88 ± 0.001 ^a^	0.84 ± 0.001 ^bc^	0.72 ± 0.001 ^d^	0.87 ± 0.01 ^ab^	0.71 ± 0.01 ^d^
ΣBCAA	0.526 ± 0.004 ^d^	1.929 ± 0.03 ^a^	1.722 ± 0.022 ^b^	1.481 ± 0.022 ^c^	1.432 ± 0.022 ^c^	1.653 ± 0.026 ^b^	1.620 ± 0.030 ^b^
ΣIAA	1.909 ± 0.01 ^e^	4.666 ± 0.02 ^a^	3.993 ± 0.02 ^c^	3.994 ± 0.02 ^c^	3.684 ± 0.02 ^d^	4.692 ± 0.02 ^a^	4.462 ± 0.03 ^b^

ΣBCAA = The sum of the concentration of the three branched chain amino acids Val, Leu, Ile. ΣIAA = The sum of the concentration of the amino acids Thr, Val, Ile, Leu, Met, Phe, Trp, Lys, His, Arg. C: casein; LP: low protein; S: soy protein; B: black bean protein; Cr: corn protein; BCr: black bean protein + corn protein; SCr: Soy protein + corn protein. Different letter superscript indicates significant differences among groups, *p* < 0.05, a > b > c.

## Supplementary Materials

Supplementary materials can be found at http://www.mdpi.com/2072-6643/8/9/573/s1. Figure S1: Western blot analysis of the (A) total mTORC1and phosphorylation of mTORC1, (B) total S6K1 and phosphorylation of S6K1 (C) total eIF4G in the muscles of rats fed different types of proteins for one day after a protein restricted period, Table S1: Amino acid composition of dietary protein sources, Table S2: primers sequence.
