# Supplementary material for: The mTORC1-Signaling Pathway and Hepatic Polyribosome Profile Are Enhanced after the Recovery of a Protein Restricted Diet by a Combination of Soy or Black Bean with Corn Protein
Source: Nutrients. 2016 Sep 20;8(9):573. doi: 10.3390/nu8090573 (PMC5037558; doi:10.3390/nu8090573)
Supplement: Supplementary file 1 [file nutrients-08-00573-s001.docx]

Supplementary Materials: The mTORC1-Signaling Pathway and Hepatic Polyribosome Profile Are Enhanced after the Recovery of a Protein Restricted Diet by a Combination of Soy or Black Bean with Corn Protein

Claudia C. Márquez-Mota, Cinthya Rodriguez-Gaytan, Pauline Adjibade, Rachid Mazroui, Amanda Gálvez, Omar Granados, Armando R. Tovar and Nimbe Torres

**Figure S1.** Western blot analysis of the (**A**) total mTORC1and phosphorylation of mTORC1; (**B**) total S6K1 and phosphorylation of S6K1 (**C**) total eIF4G in the muscles of rats fed different types of proteins for one day after a protein restricted period.

**Table S1.** Amino acid composition of dietary protein sources.

| **Amino Acids** | **FAO** | **C** | **S** | **B** | **BCr** | **SCr** | **P** | **Sp** | **Se** | **Cr** |
| --- | --- | --- | --- | --- | --- | --- | --- | --- | --- | --- |
|  | **mg/g Protein** | | | | | | | | | |
| Aspartic acid |  | 74.0 | 115.7 | 151.8 | 108.4 | 100.2 | 139.6 | 62 | 92.0 | 76.5 |
| Glutamic acid |  | 224.7 | 187.8 | 164.5 | 187.7 | 214.1 | 202.7 | 91.8 | 224.5 | 254.3 |
| Serine |  | 58.9 | 55.6 | 86.3 | 69.7 | 60.9 | 65.9 | 32.9 | 55.4 | 69.1 |
| Histidine | 19 | 26.6 | 24.8 | 29.9 | 24.3 | 24.1 | 28.5 | 10.8 | 26.7 | 23.0 |
| Glycine |  | 19.3 | 41.0 | 47.6 | 37.8 | 38.6 | 48.1 | 31.1 | 54.8 | 35.0 |
| Threonine | 34 | 44.7 | 38.1 | 59.1 | 45.2 | 39.2 | 42.1 | 31.8 | 39.0 | 40.8 |
| Arginine |  | 37.3 | 76.3 | 70.3 | 51.0 | 62.8 | 104.4 | 43.2 | 149.4 | 42.2 |
| Alanine |  | 32.0 | 43.2 | 53.8 | 68.3 | 68.1 | 51.3 | 49 | 52.4 | 106.0 |
| Valine | 35 | 56.8 | 39.0 | 57.5 | 47.8 | 42.1 | 49.3 | 31.7 | 40.8 | 46.7 |
| Methionine + cysteine | 25 | 29.4 | 23.2 | 20.7 | 28.7 | 33.0 | 18.3 | 19.9 | 53.0 | 47.9 |
| Phenylalanine + tyrosine | 63 | 114.1 | 91.1 | 129.2 | 116.7 | 117.1 | 106.6 | 58.4 | 97.6 | 143.1 |
| Isoleucine | 28 | 45.5 | 38.4 | 50.6 | 43.0 | 39.3 | 44.3 | 28.1 | 33.4 | 40.6 |
| Leucine | 66 | 95.6 | 77.0 | 104.4 | 130.4 | 122.6 | 94.9 | 54.3 | 72.5 | 192.0 |
| Lysine | 58 | 77.7 | 61.1 | 78.1 | 47.2 | 44.7 | 81.3 | 27.4 | 24.5 | 19.8 |
| Proline |  | 116.4 | 54.1 | 49.9 | 73.8 | 78.4 | 52.1 | 24.4 | 41.3 | 115.3 |
| Tryptophan | 11 | 12.4 | 12.0 | 15.6 | 10.3 | 10.3 | 10.2 | 6.9 | 16.4 | 7.6 |
| Chemical score |  | 100 | 93 | 82 | 81 | 77 | 73 | 47 | 42 | 34 |

C, casein; S, soy protein; B, black bean protein; BCr, black bean protein—corn protein; SCr, soy protein—corn protein; P, pea protein; Sp, spirulina protein; Se, sesame protein; Cr, corn protein.

**Table S2.** Primer sequences.

| **TaqMan Fluorogenic Assays** | |
| --- | --- |
| SREBP1C | *Rn01495769_m1* |
| FASN | *Rn00569117_m1* |
| SLC38A2 (SNAT2) | *Rn00710421_m1* |
| ACTIN | *Rn00667869_m1* |
| **SYBR^®^ Premix Assay** | |
| mTOR_F | GAGATGAGGAAGTGGGTGGA |
| mTOR_R | GCAGCACTTCAAGCAGAGTG |
| Rps6ka2_F | ATACCCCCGAGGAGATTCTG |
| Rps6ka2_R | AGATGGACGTCCTGTCTGCT |
| EIF4g1_F | CTGTGGGTGACCTTCTCGAT |
| EIF4g2_R | AGTCCCAGGTTTCCTCTGCT |
| rActinaB_F | CCTCTTTGCATGTCTCACTC |
| rActinaB_R | AATGTCACGCACGATTTCC |
